# DNA Methylation and Breast Cancer Risk: An Epigenome-Wide Study of Normal Breast Tissue and Blood

**DOI:** 10.3390/cancers12113088

**Published:** 2020-10-23

**Authors:** Kaoutar Ennour-Idrissi, Dzevka Dragic, Elissar Issa, Annick Michaud, Sue-Ling Chang, Louise Provencher, Francine Durocher, Caroline Diorio

**Affiliations:** 1 Département de Médecine Sociale et Préventive, Faculté de Médecine, Université Laval, Québec, QC G1V 0A6, Canada; kaoutar.ennour-idrissi.1@ulaval.ca (K.E.-I.); dzevahira-dzevka.dragic.1@ulaval.ca (D.D.); 2 Centre de Recherche sur le Cancer, Centre de Recherche du CHU de Québec-Université Laval, Québec, QC G1R 3S3, Canada; elissar.issa.1@ulaval.ca (E.I.); Annick.Michaud@crchudequebec.ulaval.ca (A.M.); Sue-Ling.Chang@crchudequebec.ulaval.ca (S.-L.C.); francine.durocher@crchudequebec.ulaval.ca (F.D.); 3 Département de Biologie Moléculaire, de Biochimie Médicale et de Pathologie de l’Université Laval, Québec, QC G1V 0A6, Canada; 4 Département de Médecine Moléculaire, Faculté de Médecine, Université Laval, Québec, QC G1V 0A6, Canada; 5 Centre des Maladies du sein du CHU de Québec-Université Laval, Québec, QC G1S 4L8, Canada; Louise.Provencher@chudequebec.ca

**Keywords:** breast cancer risk, DNA methylation, epigenome-wide, case-control study

## Abstract

**Simple Summary:**

As known breast cancer risk factors do not accurately predict the risk of developing breast cancer, breast cancer screening is still based solely on age. At the interface between environmental exposures and gene expression, DNA methylation patterns are potential biomarkers for assessing breast cancer risk, thus allowing for implementation of personalized screening and risk-reducing strategies. We used a comprehensive high-throughput DNA methylation assay in an unprecedented study design of normal breast epithelial tissue to detect methylation changes that are causally related to breast cancer occurrence and replicated our analyses in two independent datasets of normal breast tissue and blood. We identified several methylation differences in cancer-related genes, some of which overlapped between normal breast tissue and blood and were reported in previous studies. Our findings warrant further investigation on novel biomarkers for identifying women that will benefit the most from breast cancer screening.

**Abstract:**

Differential DNA methylation is a potential marker of breast cancer risk. Few studies have investigated DNA methylation changes in normal breast tissue and were largely confounded by cancer field effects. To detect methylation changes in normal breast epithelium that are causally associated with breast cancer occurrence, we used a nested case–control study design based on a prospective cohort of patients diagnosed with a primary invasive hormone receptor-positive breast cancer. Twenty patients diagnosed with a contralateral breast cancer (CBC) were matched (1:1) with 20 patients who did not develop a CBC on relevant risk factors. Differentially methylated Cytosine-phosphate-Guanines (CpGs) and regions in normal breast epithelium were identified using an epigenome-wide DNA methylation assay and robust linear regressions. Analyses were replicated in two independent sets of normal breast tissue and blood. We identified 7315 CpGs (FDR < 0.05), 52 passing strict Bonferroni correction (*p* < 1.22 × 10^−7^) and 43 mapping to known genes involved in metabolic diseases with significant enrichment (*p* < 0.01) of pathways involving fatty acids metabolic processes. Four differentially methylated genes were detected in both site-specific and regions analyses (*LHX2, TFAP2B, JAKMIP1, SEPT9*), and three genes overlapped all three datasets (*POM121L2, KCNQ1, CLEC4C*). Once validated, the seven differentially methylated genes distinguishing women who developed and who did not develop a sporadic breast cancer could be used to enhance breast cancer risk-stratification, and allow implementation of targeted screening and preventive strategies that would ultimately improve breast cancer prognosis.

## 1. Introduction

As a major and increasing worldwide public health burden, breast cancer prompts the need for identification of early breast tissue molecular alterations that could be used for risk-tailored early diagnostic and effective primary prevention strategies. DNA methylation, a covalent addition of a methyl group to Cytosine-phosphate-Guanine (CpG) dinucleotides, summarizes genetic, environmental, and stochastic events that contribute to inter-individual variation in gene expression and ultimately, to variation in common complex diseases risk such as breast cancer [1,2]. In fact, widespread DNA methylation alterations in normal breast tissue adjacent to cancer that become enriched with breast cancer progression have been identified, suggesting that DNA methylation alterations predate the emergence of breast cancer [3].

Demonstrating a mechanistic link between DNA methylation patterns and breast cancer occurrence remains a considerable challenge due to the cell type specificity of DNA methylation and the cell type heterogeneity of examined tissues. Such a mechanistic link should be supported by the identification of tissue-specific DNA methylation changes in normal breast tissue prior to breast cancer occurrence [1]. In fact, many DNA methylation studies have been conducted on blood samples, and the detected methylation marks were not consistent across studies, even when these marks were validated and reproduced in independent datasets within the same study. These inconsistencies can mainly be explained by methodological biases [4] but can also suggest some limitations when considering blood samples for detecting methylation marks of tissue-specific cancers. The only feasible approach used to date to identify causative molecular alterations in normal breast tissue is the comparison of normal tissue from healthy individuals to normal tissue adjacent to cancer. As few as three studies have attempted this approach for the detection of methylation marks in normal breast tissue [4]. However, this approach is compromised by the existence of cancer field effects [3]. In fact, genetic and epigenetic field effects in histologically normal-appearing tissue adjacent to cancer have been reported as far as 4 cm from primary breast tumors [5,6]. These molecular alterations may reflect both precancerous alterations that led to breast cancer development and alterations induced by the microenvironment of the adjacent developing cancer [7]. 

To cancel out field effects secondary to the cancer environment, we used a nested case–control design to compare the normal breast tissue adjacent to primary tumors between breast cancer patients who developed a contralateral breast cancer (i.e., a second primary breast cancer in the opposite breast) to those who did not develop a contralateral breast cancer. The rationale behind this design is that both breasts of the same patient presumably bear the same precancerous DNA methylation alterations that summarize the complex interplay between genetic and environmental factors associated with her individual risk of developing a primary breast cancer in either breast. To further confirm that the detected DNA methylation alterations predate a second primary breast cancer occurrence, we replicated our analyses in two independent sets of case–control pairs, in which DNA samples of normal breast tissue and blood were obtained before or at the time of a first primary breast cancer occurrence.

## 2. Materials and Methods 

### 2.1. Study Design and Population

We conducted a nested case–control study based on a cohort of 757 patients diagnosed between 2000 and 2007 with a primary invasive hormone receptor-positive and non-metastatic breast cancer at a breast cancer reference center, the “Centre des maladies du sein du CHU de Québec”. Biological characteristics of tumors were extracted from pathology reports. Demographic and clinical data collected at diagnosis were extracted from medical records and entered into a database by trained nurses and registrars. Women were eligible if they had no previous diagnosis of cancer other than non-melanoma skin cancer and did not receive any treatment prior to surgery. Using an incidence density sampling scheme, 20 patients diagnosed with a contralateral breast cancer (in situ or invasive) at least 12 months after their first breast cancer (cases) were matched (1:1) with 20 patients who did not develop a contralateral breast cancer (controls). Matching variables were the year of surgery (±2 years), age (±5 years), menopausal status, family history of breast cancer (yes/no), histologic type (ductal vs. lobular) of the primary tumor, human epidermal growth factor receptor 2 (HER2) status of the primary tumor, and hormone therapy (yes/no). For cases and controls, normal breast tissue was collected from breast surgery specimens. 

Two additional sets of case–control pairs were used to select differentially methylated sites that predate a second primary breast cancer occurrence. The first set consisted of four breast cancer patients diagnosed with a first invasive hormone receptor-positive and non-metastatic breast cancer (cases) drawn from the same cohort described above, and four women diagnosed with a benign breast lesion (controls) drawn from the tissue biobank of the “Centre des maladies du sein du CHU de Québec”. Women were matched (1:1) for the year of surgery (±2 years) and age (±5 years). For cases and controls, normal breast tissue was collected from breast surgery specimens prior to any other treatment. 

The second set consisted of six women with high mammographic density (>65%) who eventually developed an invasive breast cancer (cases), and six women (controls) with low mammographic density (<15%) who did not develop a breast cancer by the time their matched case developed a breast cancer. These women were drawn from a cohort of 737 women who attended a mammography screening at the “Clinique radiologique Audet” (Québec, QC, Canada) between February and December 2001 [8]. Anthropometric data (weight and height measures) were measured at enrollment by a qualified research nurse, the women’s characteristics were collected using standardized questionnaires administered by telephone interview, and clinical data were extracted from medical records. Cases and controls were matched (1:1) for age (±3 years), family history of breast cancer (yes/no), body mass index (BMI, 18.5 to <25; 25 to <30), number of full-term pregnancies, and breast biopsy (yes/no). For these women, blood was collected at the time of mammography 5.6 ± 1.7 years on average before breast cancer occurrence for cases (median = 5.5 years, range 3.4 to 8.1 years). 

All participants provided written informed consent. The study protocol was reviewed and approved by the research ethics committee of the CHU de Québec-Université Laval Research Center. The data that support the findings of this study are available upon reasonable request from the corresponding author (C.D.). The data are not publicly available due to legal restrictions to respect research participant privacy and consent.

The design of the main analysis aimed at identifying differentially methylated CpGs sites in normal breast epithelium that are causally associated with breast cancer occurrence, i.e., while canceling out cancer field effects, by comparing normal breast tissue of two groups of patients both exposed to similar cancer field effects. The rationale behind this design is that once cancer field effect has been canceled out from the affected breast, normal breast epithelium both from the affected breast and the non-affected contralateral breast harbor the same epigenetic marks for each individual patient, because both breasts have been exposed to the same genetic and environmental factors. In other words, this study design is comparable to having sampled the unaffected breast (i.e., the contralateral breast) and followed-up patients for the development of a primary breast cancer in this unaffected contralateral breast. Matching for factors that are known to be associated with contralateral breast cancer occurrence ensured that the setting is similar to obtaining normal breast tissue from women who had never had a previous breast cancer and comparing women who develop a primary breast cancer to those who do not develop a primary breast cancer. 

Beyond a simple validation of our findings in the exact same study design, we decided to select differentially methylated CpGs sites that replicate in a more traditional (but less robust because of confounding by cancer field effects) study design. In the secondary dataset #1, we compared women who have developed a first primary breast cancer to women who had not developed a first primary breast cancer, which is the setting “artificially” created by the robust design of the main dataset.

Finally, the rationale behind the choice of the secondary dataset #2 was to select those differentially methylated CpGs that could also be detected in blood-derived DNA, i.e., those methylation marks that may have been induced early during development and propagated soma-wide and that could be useful as non-invasive biomarkers for breast cancer screening. Here, we compared women who developed a primary breast cancer with women who did not develop a primary breast cancer, by using blood samples prospectively collected several years before breast cancer occurrence in cases. These women had a mammography screening at time of blood collection, and those who developed breast cancer during follow-up had higher breast density at baseline than those who did not develop a breast cancer during follow-up (breast density is a known risk factor for breast cancer occurrence).

Thus, our three datasets compared women who developed a breast cancer (first primary or second primary breast cancer) to women who did not develop breast cancer, using three different strategies.

### 2.2. DNA Methylation Measurement

For breast tissue samples, normal breast epithelium located at least 1.0 cm from the primary tumor of cases and controls was identified on corresponding hematoxylin-eosin (H&E) stained slides. Ten to fifteen cores of 1.0 mm with at least 75% epithelial cells content were extracted from formalin-fixed paraffin-embedded (FFPE) tissue blocks and were used to build a tissue microarray (TMA) block for each patient. TMA blocks were serially sectioned at 10.0 μm. H&E-stained histologic tissue sections were verified for cellular content in the first, every 10th, and in the last section. A column-based method for DNA extraction from TMA sections of each patient was performed using GeneRead DNA FFPE deparaffinization solution (Qiagen, Mississauga, Ontario, ON, Canada) and the QIAamp DNA FFPE kit (Qiagen, Mississauga, Ontario, ON, Canada) for subsequent extraction steps. Deparaffinization was done twice to ensure complete paraffin elimination and proteinase K was incubated at 56 °C in ATL buffer for three days with 20 µL of proteinase K added each 24 h.

For blood samples, DNA was extracted from buffy coats using the Gentra Puregene DNA extraction kit (QIAGEN Inc., Canada) following the manufacturer’s protocol.

Quantification of DNA methylation was carried out at McGill University and Génome Québec Innovation Centre (Montreal, Quebec, QC, Canada) using the Infinium Human Methylation 450K (HM450k) BeadChip (Illumina Inc., San Diego, CA, USA), after bisulfite conversion, Infinium FFPE quality control, and DNA restoration, according to the manufacturer’s instructions. The HM450k has been extensively validated and provides reliable coverage of 485,512 CpG sites across 99% of RefSeq genes and 96% of CpG islands in the human genome [9]. In order to test for potential batch effects, eight samples were replicated between or within batches. Hybridized and processed arrays were scanned using Illumina iScan (Illumina Inc., San Diego, CA, USA) to produce. IDAT files with raw probe intensities.

### 2.3. Data Preprocessing and Statistical Analyses

Raw methylation data preprocessing and statistical analyses were performed using R software version 3.6.2 [10] and Bioconductor packages [11]. The same preprocessing steps were performed separately for breast tissue samples and blood samples.

Data from IDAT files were read using the minfi package [12]. Quality control plots for bisulfite conversion, extension, and hybridization were generated using the minfi and ENmix [13] packages. Probes that failed in one or more samples based on detection *p*-value > 0.01, probes overlapping a CpG site or single-base extension of the measured methylation loci, cross-reactive probes [14], probes with multimodal methylation distributions identified using ENmix package [13], and probes from the X and Y chromosomes were filtered out. Probes from the X chromosome were excluded to avoid a higher probability of both type 1 and type 2 errors associated with analyses of data from sex chromosomes, compared to autosomal data [15].

The data-driven separate normalization method from the wateRmelon package was used for background adjustment and between-array normalization [16]. The regression on correlated probes method from the ENmix package was used for probe type bias adjustment [17]. Using multidimensional scaling (MDS) plots, no obvious batch, chip, or slide effect was detected and no outlier sample was identified. Intra- and inter-batch samples (n = 4) were then removed from the analyses. Beta-values were logit-transformed into M-values for statistical analyses. Of the 485,512 CpG sites included on the array, 409,741 autosomal CpG sites were included in the analyses of breast tissue samples (Figure S1) and 429,014 in the analyses of blood samples (Figure S2). In total, 40 samples (20 case-control pairs) were included in the main analysis of normal breast tissue samples (patients who developed a contralateral breast cancer vs. patients who did not develop a contralateral breast cancer). Eight samples (four case-control pairs) were included in the secondary analysis of normal breast tissue samples (patients with a primary breast cancer vs. patients with a benign breast tumor) and twelve samples (six case-control pairs) were included in the analysis of blood samples (women who developed a breast cancer vs. women who did not develop a breast cancer).

Global methylation differences between cases and controls for each set were compared using the Wilcoxon signed-rank test and mean beta-values, both globally and by CpG island and gene spatial distribution.

Probe-wise differential methylation analysis using M-values was performed with the limma package robust linear models. Robust empirical Bayes method was used to generate moderated paired t-test statistics and associated p-values for each CpG site. For blood samples, white blood cell type proportions were estimated using the method described by Houseman et al. [18] within the minfi package. Confounding by cell type proportions was identified, and differentially methylated CpGs associated with cell type proportions were excluded from further analyses. To determine biological plausibility, differentially methylated CpGs with Benjamini and Hochberg-adjusted *p*-values (FDR *q*-values) < 0.05 were selected for functional annotations (Gene Ontology, KEGG) and were analyzed using the gometh function of the missMethyl package [19]. CpG sites passing the FDR threshold in the main nested case-control analysis of normal breast tissue samples were considered replicated in the two secondary sets (normal breast tissue and blood samples) if the nominal p-value in these secondary analyses was < 0.05 with the same direction of association. CpG sites passing the strict Bonferroni correction (nominal *p*-value < 1.22 × 10^−7^) in the main analysis and those replicated in the secondary analyses were further compared to the CPGA SAGE database, using the Database for Annotation, Visualization and Integrated Discovery (DAVID) v6.8 to select differentially methylated genes for which significant methylation changes are consistent with their differential expression in breast cancer.

To identify concordant differentially methylated regions of several consecutive CpG sites (distance to the next consecutive site less than 1000 nucleotides), differential methylation analysis of regions with Benjamini and Hochberg correction for multiple comparisons was performed using the DMRcate package [20]. Regions with Stouffer *p*-value < 0.05, maximum difference > 0.05, and containing at least two or more CpG sites were selected. Genes identified by the region approach were compared to those identified by the individual CpG site approach.

## 3. Results

### 3.1. Participants Characteristics

The 40 patients included in the main analysis were aged between 33 and 69 years (mean ± standard deviation (SD, 51.0 ± 8.2), were mainly postmenopausal, and 75% reported a family history of breast cancer. They had stage I to III estrogen receptor (ER)-positive invasive breast carcinomas. All received hormone therapy and 70% received chemotherapy. Their characteristics, according to case or control status, are summarized in Table 1. Distribution of contralateral breast cancer risk factors was similar for cases (patients who developed a contralateral breast cancer) and controls (patients who did not develop a contralateral breast cancer). There were more grade 3 primary breast cancers among controls, whereas cases received radiotherapy slightly more often. These two factors are not known to be associated with contralateral breast cancer risk [21,22]. 

The eight patients included in the secondary analysis of normal breast tissue were aged between 47 and 66 years (mean ± SD, 55.5 ± 7.0), were mainly postmenopausal, and 63% reported a family history of breast cancer. Their characteristics, according to case or control status, are summarized in Table S1. Cases were less likely to have a family history of breast cancer than controls and had stage I to II invasive ER-positive breast carcinomas, all of which received hormone therapy and 50% received chemotherapy.

The twelve women included in the secondary analysis of blood samples were aged between 38 and 53 years (mean ± SD, 43.6 ± 4.7), were all premenopausal, and 16.7% reported a family history of breast cancer. Their characteristics, according to case or control status, are summarized in Table S2. Cases were more likely to have breastfed their children. Cases developed an invasive breast carcinoma 6.0 ± 1.6 years on average after their blood sample, for which 33.3% received chemotherapy and 66.7% received hormone therapy. They were aged between 45 and 62 years at diagnosis (mean ± SD, 50.0 ± 6.1) and 16.7% of them were postmenopausal.

### 3.2. Global Methylation and Breast Cancer Risk

There was no global methylation difference between cases and controls, both for average methylation across all included CpGs sites and when CpGs were grouped by island location or by gene region (Table 2, Tables S3 and S4).

### 3.3. Individual CpG Sites and Breast Cancer Risk

Of the 409,741 included CpGs in the main analysis of normal breast tissue, 7315 CpGs were differentially methylated at FDR *q*-values < 0.05, of which 954 CpGs had a *q*-value < 0.01 (Table S5). Functional annotation analyses indicated that genes related to these 7315 differentially methylated CpGs are mainly enriched in pathways involving epithelial cells (Table S6). Fifty-two CpGs passed the strict Bonferroni correction (*p*-value < 1.22 × 10^−7^). These 52 CpGs were predominantly located in CpGs islands and gene body regions, and 56% were hypomethylated. Forty-three of them were mapped to known reference genes involved in metabolic diseases. Pathway analysis of these 43 genes identified six enriched pathways (*p*-value < 0.01) involving fatty acids metabolic processes (Table S7). One gene, LHX2, harbored significant methylation changes at two different CpG positions. Of the 43 genes, 24 are known to be enriched in breast cancer tissues, of which 9 are also enriched in normal breast epithelium (Table 3), and 15 genes, namely *ELOVL6, LHX2, CLIC6, CAMK2N1, FAT1, TFAP2B, HOXA11, TOLLIP, SEPT9, JAKMIP1, CAND2, PODNL1, C12orf41 (KANSL2), GLS2,* and *NCOR1*, harbored significant methylation changes consistent with their differential expression in breast cancer.

Of the 409,741 included CpGs in the secondary analysis of normal breast tissue, 56,510 CpGs were differentially methylated at a nominal *p*-value < 0.05, of which 15,505 CpGs had a nominal *p*-value < 0.01. None were differentially methylated at an FDR *q*-value < 0.05 (Table S8).

Of the 429,014 included CpGs in the secondary analysis of blood tissue, after exclusion of CpGs associated with cell type composition, 29,526 CpGs were differentially methylated at a nominal *p*-value < 0.05, of which 8707 CpGs had a nominal *p*-value < 0.01. Fifty-nine CpGs were differentially methylated at an FDR q-value < 0.05, of which six CpGs had a *q*-value < 0.01 (Table S9). Of these, five CpGs passed the strict Bonferroni correction (*p*-value < 1.22 × 10^−7^), of which three were mapped to known reference genes (Table 4). 

Of the 7315 CpG sites passing the FDR of 0.05 in the main analysis of normal breast tissue, 86 CpGs were also differentially methylated in both secondary analyses (normal breast tissue and blood samples) at a nominal *p*-value < 0.05 (Table S10). Of these, 9 CpGs were differentially methylated in the same direction of association between the main analysis and the secondary analysis of normal breast tissue, 34 CpGs were differentially methylated in the same direction of association between the main analysis and the secondary analysis of blood tissue, and 6 CpGs had the same direction of association in all three datasets (Table 5). Of these, three genes, POM121L2, KCNQ1, and CLEC4C, harbored significant methylation changes consistent with their differential expression in breast cancer.

### 3.4. Differential Methylation of Regions and Breast Cancer Risk

There were 1105 differentially methylated regions (DMRs) associated with breast cancer risk in the main analysis of normal breast tissue (Table S11). Pathway analysis of genes associated with these 1105 DMRs identified three enriched pathways (*p*-value = 0.0003) involving negative regulation of cholesterol/sterols biosynthetic processes (Table S12). Ninety-six DMRs had a Stouffer *p*-value < 0.05, a maximum difference > 0.05, and contained two or more CpG sites (Table S11). The 20 most significant DMRs are presented in Table 6. 

Of the 43 differentially methylated genes associated with breast cancer risk in the individual CpG sites analysis of normal breast tissue main analysis, *LHX2* was included in three DMRs, *TFAP2B* in two DMRs, *JAKMIP1* in one DMR, and *SEPT9* in one DMR. 

## 4. Discussion

The present study aimed at identifying normal breast tissue methylation patterns that may predispose to breast cancer development, using a robust study design unprecedented in previous breast cancer epigenome-wide association studies. To cancel out field effects, our main nested case–control analysis compared normal breast tissue adjacent to primary tumors of breast cancer patients who developed and those who did not develop a contralateral breast cancer. We identified 7315 individual CpG sites with an FDR *q*-value < 0.05 and 52 CpG sites at the strict Bonferroni nominal *p*-value < 1.22 × 10^−7^, of which 43 were mapped to known genes involved in metabolic diseases. Pathway analysis of these 43 distinct genes identified six enriched pathways (*p*-value < 0.01) involving fatty acids metabolic processes.

One gene, *LHX2*, harbored significant methylation changes at two different CpG positions, while 15 genes harbored significant methylation changes consistent with their differential expression in breast cancer. Of these, *LHX2, TFAP2B, JAKMIP1,* and *SEPT9* were also included in significantly differentially methylated regions. The *LHX2* gene codes for the LIM homeobox 2 protein, a transcription factor downstream of p63 and NF-κB, and upstream of Wnt/β-catenin, Bmp, and Shh [23], that has a critical role during the epithelial–mesenchymal transition in normal and cancerous breast epithelial cells [24]. This gene has been shown to harbor aberrant methylation in primary breast tumors [25]. The *TFAP2B* gene codes for the transcription factor AP-2 beta, a sequence-specific DNA-binding protein that has been recognized as an oncogene that mediates cancer cell proliferation, apoptosis, invasion, and migration via the COX-2 signaling pathway in vitro and in vivo [26]. TFAP2B is also expressed in breast tissue, where it is thought to coordinate HER2 and ER [27] and has been associated with breast cancer prognosis [28]. The *JAKMIP1* gene codes for the Janus kinase and microtubule interacting protein 1 and has been shown to be highly expressed in tumor samples, where it enhances the proliferation of cancer cells [29]. Its upregulation affects cell proliferation via the Wnt and beta-catenin pathways [29]. The *SEPT9* gene codes for Septin 9, a protein involved in cytokinesis and cell cycle control that has been implicated in early breast cancer development [30]. The *SEPT9* gene methylation has been detected in breast cancer tissue [31].

To further detect DNA methylation alterations that predate a second primary breast cancer occurrence, we used two independent sets of case–control pairs in which DNA samples of normal breast tissue and blood were obtained before a second breast cancer occurrence. Out of the 7315 individual CpG sites identified in the main nested case–control analysis, six CpG sites were also differentially methylated with the same direction of association in both secondary sets’ analyses, of which five mapped to known reference genes. Of these, three genes, namely *POM121L2*, *KCNQ1*, and *CLEC4C*, harbored significant methylation changes consistent with their differential expression in breast cancer. The *POM121L2* gene codes for POM121 transmembrane nucleoporin like 2, which has been shown to be upregulated in triple negative breast cancer [32]. *KCNQ1* codes for the potassium voltage-gated channel subfamily Q member 1, which has been shown to play important physiological roles in the mammary epithelium [33] and has been suggested to act as a tumor suppressor and regulator of the epithelial–mesenchymal transition in colorectal cancers [34,35]. *CLEC4C* codes for a lectin-type cell surface receptor that may play a role in antigen capturing by dendritic cells, inflammation, and immune response, and has been shown to be upregulated in triple negative breast cancer [36].

Many epigenome-wide studies have investigated the association between DNA methylation and breast cancer risk using blood-derived DNA and the HM450k BeadChip, while as few as three studies measured breast tissue DNA methylation [4]. These studies identified between 0 and 2761 differentially methylated CpGs, with none of the identified differentially methylated sites overlapping between these studies, and suffered major methodological issues, especially pertaining to incomplete control of confounding and suboptimal preprocessing methods [4]. Nevertheless, four of our detected differentially methylated CpGs in the main analysis were also differentially methylated in the same direction of association (all hypomethylated in breast cancer) in previous epigenome-wide studies, namely cg07180460 (*ZSWIM6*), cg22731164 (*GPR176*), and cg18726036 (*FKBP5*) in a study of blood DNA methylation from the Sister Study [37], and cg02168584 *(DLX2-AS1*) in a study of genetically predicted DNA methylation of patients from the Breast Cancer Association Consortium [38], all of which have been shown to be dysregulated in breast cancer cell lines [39,40,41,42].

Taken together, our findings support the hypothesis that detectable methylation differences in cancer-related genes in normal breast tissue predate the occurrence of breast cancer. Some of these methylation changes were also detectable in blood DNA, suggesting that these methylation changes may have been induced early during development and propagated soma-wide [2,43], and could be useful as biomarkers for non-invasive screening to identify women with increased risk of developing breast cancer. Methylation changes that were specific to normal breast tissue may have occurred during adulthood as a result of ageing and lifetime exposure to known and unknown risk factors [2,43], and could be useful for identifying these unknown risk factors and for potential targeted interventions based on epigenetic agents to prevent breast cancer occurrence [1].

Using an original and novel study design, we were able to assess methylation changes in normal breast epithelial tissue while minimizing the risk of confounding by cancer field effects. The main strengths include the use of conventional epidemiological approaches to control for selection bias (nested case–control design) and confounding bias (matching for breast cancer risk and prognostic factors), two important drawbacks in previous epigenome-wide DNA methylation studies of breast cancer [36]. We used *a priori*, up-to-date, and recommended data preprocessing methods and workflow, which prevent inflation of the false-positive rate resulting from data-driven selection of preprocessing methods. In addition, we conducted both site-specific and DMR analyses, and we replicated the analyses in two independent datasets. The main limitation of the study is the relatively small sample size, which could have limited the detection of genuine methylation differences (i.e., low study power). However, by using the appropriate data preprocessing methods coupled with the doubly robust statistical modeling approach, which minimizes the risk of false-negative rate, we were able to detect more differentially methylated CpG sites than larger studies [36].

While robust and promising, our results need to be validated in other populations and with other DNA methylation measurement methods. Epigenome-wide DNA methylation methods are particularly suitable for hypothesis generation as they capture the dynamics of several sites simultaneously across the entire genome, thus being less prone to bias than candidate gene methylation studies [44]. The next step would be to validate the differentially methylated sites and related genes detected by these methods using a different measurement method, such as a PCR-based method, in a candidate-gene methylation study. A transcriptional or protein expression analysis should then be performed to confirm the functional impact of the detected methylation differences and its association with breast cancer occurrence [45].

## 5. Conclusions

We identified four breast cancer risk-related genes that are differentially methylated in both site-specific and DMR analyses (*LHX2, TFAP2B, JAKMIP1,* and *SEPT9*) in the main analysis of normal breast tissue, and three genes overlapping the main analysis and two independent datasets of normal breast tissue and blood (*POM121L2, KCNQ1,* and *CLEC4C*). No significant global methylation differences were observed between cases and controls in any of the three datasets. Once validated, our identified genes could be used to enhance risk stratification for prevention of breast cancer and for developing new strategies for primary breast cancer prevention and treatment.

## Figures and Tables

**Table 1:** Characteristics of patients who developed (cases) and who did not develop (controls) a contralateral breast cancer, in the main analysis of normal breast tissue.

Characteristics *	Cases (n = 20)	Controls (n = 20)
Age (years)		
Mean ± SD	50.9 ± 8.1	51.1 ± 8.4
Median [range]	51.5 [33.0–67.0]	51.0 [33.0–69.0]
Postmenopausal	12 (60%)	12 (60%)
Family history of breast cancer (yes)	15 (75%)	15 (75%)
Parity (yes)	11 (55%)	15 (75%)
Age at first pregnancy (years)		
Mean ± SD	25.0 ± 3.2	27.0 ± 6.2
Median [range]	24.0 [21.0–31.0]	26.0 [19.0–40.0]
Breastfeeding (yes)	6 (30%)	7 (35%)
Ever smokers	12 (60%)	7 (35%)
Alcohol consumption (yes)	14 (70%)	11 (55%)
Body mass index (kg/m^2^)		
Mean ± SD	25.3 ± 5.5	23.2 ±3.5
Median [range]	23.6 [16.3–41.0]	22.0 [18.7–32.9]
Histologic type		
Ductal, invasive	19 (95%)	19 (95%)
Lobular, invasive	1 (5%)	1 (5%)
Positive lymph nodes		
0	12 (60%)	12 (60%)
1–3	6 (30%)	6 (30%)
≥4	2 (10%)	2 (10%)
Grade		
1	8 (40%)	3 (15%)
2	9 (45%)	10 (50%)
3	1 (5%)	6 (30%)
Stage		
I	8 (40%)	8 (40%)
II	11 (55%)	9 (45%)
III	1 (5%)	3 (15%)
ER status		
Negative	0	0
Positive	20 (100%)	20 (100%)
HER2 status		
No evaluation	4 (20%)	4 (20%)
Negative	15 (75%)	15 (75%)
Positive	1 (5%)	1 (5%)
Chemotherapy	14 (70%)	14 (70%)
Radiotherapy	18 (90%)	16 (80%)
Hormone therapy	20 (100%)	20 (100%)

* at time of diagnosis of the primary breast cancer; n = number; SD—standard deviation.

**Table 2:** Mean methylation beta-values of patients that developed a contralateral breast cancer (cases) and patients who did not develop a contralateral breast cancer (controls), in the main analysis of normal breast tissue.

	Number of CpGs	Cases (n = 20)	Controls (n = 20)	*p*-Value *
**All Included CpGs**	409,741	0.516	0.516	0.784
**Distribution relative to island**				
Island	134,756	0.278	0.278	0.841
N_Shelf	19,957	0.729	0.729	0.648
N_Shore	53,628	0.506	0.506	0.571
OpenSea	141,767	0.694	0.694	0.756
S_Shelf	17,738	0.734	0.734	0.648
S_Shore	41,895	0.495	0.494	0.701
**Distribution relative to gene**				
TSS1500	72,345	0.383	0.383	0.784
TSS200	55,782	0.246	0.246	0.189
5’UTR	57,346	0.356	0.356	0.621
1stExon	34,812	0.263	0.263	0.177
Body	150,226	0.626	0.626	0.571
3’UTR	16,606	0.712	0.713	0.927
Intergenic	94,333	0.604	0.605	0.898
Promoter	83,922	0.181	0.180	0.154

* Wilcoxon signed-rank test; TSS—transcription start site; CpG—Cytosine-phosphate-Guanine.

**Table 3:** CpG sites associated with breast cancer risk in the main analysis of normal breast tissue and passing strict Bonferroni correction (*p*-value < 1.22 × 10^−7^).

CpG	Chr	Gene	Gene Region	CpG Island Region	logFC	*p*-Value
cg03835987	chr4	ELOVL6 *	TSS1500	S_Shore	−0.546	1.61 × 10^−11^
cg19462193	chr12	VPS33A	TSS200	Island	0.288	9.98 × 10^−11^
cg11051893	chr13	**MPHOSPH8**	TSS200	Island	0.337	2.45 × 10^−10^
cg08716396	chr1	**SDHC**	TSS1500	Island	0.299	4.01 × 10^−9^
cg09155575	chr17	**ORMDL3**	TSS200	Island	0.247	4.47 × 10^−9^
cg27160524	chr2	PXDN *	Body	OpenSea	−0.314	9.57 × 10^−9^
cg00982919	chr9	LHX2	Body	Island	−0.618	1.13 × 10^−9^
cg15295166	chr21	**CLIC6**	TSS1500	N_Shore	−0.291	1.39 × 10^−9^
cg13401911	chr1	**CAMK2N1**	1stExon;5′UTR	Island	0.334	1.64 × 10^−9^
cg14291745	chr10	-	-	N_Shore	−0.379	3.14 × 10^−9^
cg24410535	chr4	**FAT1**	TSS200	Island	−0.459	3.96 × 10^−9^
cg19066273	chr17	**-**	-	Island	−0.516	4.08 × 10^−9^
cg11031869	chr7	**-**	-	OpenSea	−0.298	4.28 × 10^−9^
cg14910241	chr3	**TGFBR2**	Body	Island	−0.221	5.98 × 10^−9^
cg13492692	chr9	LHX2	Body	Island	−0.378	7.30 × 10^−9^
cg13322131	chr6	**TFAP2B**	3′UTR	S_Shore	−0.537	7.91 × 10^−9^
cg13170891	chr6	**PPP1R10**	3′UTR	OpenSea	0.322	7.92 × 10^−9^
cg00705992	chr7	HOXA11	Body;TSS1500	N_Shore	−0.308	1.13 × 10^−8^
cg05992226	chr14	SMEK1	Body	Island	−0.254	1.21 × 10^−8^
cg23695222	chr8	-	-	OpenSea	−0.329	1.22 × 10^−8^
cg06562570	chr14	**VRK1**	TSS200	Island	0.232	1.86 × 10^−8^
cg05508048	chr13	**INTS6**	TSS200	Island	0.306	2.15 × 10^−8^
cg11170179	chr19	KLK10 *	5′UTR	Island	0.362	2.26 × 10^−8^
cg00954771	chr11	TOLLIP	Body	Island	0.307	3.04 × 10^−8^
cg01868405	chr1	**-**	-	Island	−0.427	3.10 × 10^−8^
cg14011789	chr17	SEPT9 *	Body;5′UTR	OpenSea	0.486	3.43 × 10^−8^
cg10332787	chr14	C14orf106	TSS200	Island	0.205	3.54 × 10^−8^
cg16724588	chr4	JAKMIP1	Body	Island	−0.585	3.62 × 10^−8^
cg07557790	chr3	CAND2 *	TSS1500	N_Shore	−0.391	3.78 × 10^−8^
cg05559558	chr6	KIAA1244	1stExon;5′UTR	Island	0.204	4.08 × 10^−8^
cg20430816	chr4	**STBD1**	Body	Island	−0.259	4.16 × 10^−8^
cg10092770	chr20	NCRNA00029	TSS200	S_Shore	0.365	4.40 × 10^−8^
cg05335186	chr13	-	-	N_Shore	0.285	4.48 × 10^−8^
cg14255751	chr15	PTPLAD1	TSS200	N_Shore	0.252	4.83 × 10^−8^
cg03171770	chr10	-	-	Island	−0.476	4.86 × 10^−8^
cg24354933	chr19	**PODNL1**	Body;TSS200	OpenSea	0.414	4.93 × 10^−8^
cg02457207	chr16	**SPG7**	Body	N_Shelf	−0.315	4.97 × 10^−8^
cg14047370	chr7	HECW1	Body	OpenSea	−0.461	5.23 × 10^−8^
cg01764116	chr1	**VPS45**	5′UTR;1stExon	Island	0.276	5.40 × 10^−8^
cg22606205	chr1	PKP1	5′UTR;1stExon	Island	−0.316	5.79 × 10^−8^
cg23535449	chr17	AATK	TSS200;Body	N_Shelf	0.250	6.17 × 10^−8^
cg21352943	chr12	C12orf41	TSS200	Island	−0.261	6.25 × 10^−8^
cg06380356	chr2	-	-	Island	−0.371	7.02 × 10^−8^
cg24014538	chr13	FARP1 *	Body	OpenSea	−0.405	7.15 × 10^−8^
cg10938586	chr10	**-**	-	Island	0.336	7.60 × 10^−8^
cg14319655	chr19	AKAP8	TSS200	Island	0.217	7.88 × 10^−8^
cg09103187	chr6	TRERF1 *	Body	OpenSea	−0.250	8.69 × 10^−8^
cg17939432	chr22	ZC3H7B	Body	S_Shore	0.311	8.82 × 10^−8^
cg12241297	chr5	HNRNPA0 *	TSS1500	Island	0.239	1.08 × 10^−7^
cg05295557	chr14	NKX2-1	Body;1stExon	Island	−0.249	1.14 × 10^−7^
cg20877313	chr12	GLS2	1stExon	Island	−0.326	1.21 × 10^−7^
cg00253204	chr17	NCOR1 *	1stExon;5′UTR	Island	−0.212	1.21 × 10^−7^

**In bold:** genes enriched in breast cancer (CGAP); * genes enriched in the normal epithelium (CGAP); CGAP—Cancer Genome Anatomy Project; Chr—chromosome.

**Table 4:** CpG sites associated with breast cancer risk in the secondary analysis of blood samples and passing strict Bonferroni correction (*p*-value < 1.22 × 10^−7^).

CpG	Chr	Gene	Gene Region	CpG Island Region	logFC	*p*-Value
cg13449967	chr11	**ATG2A**	Body	N_Shore	1.113	7.00 × 10^−10^
cg00876501	chr4	**EIF4E**	5’UTR;1stExon;TSS1500	S_Shore	0.896	2.22 × 10^−9^
cg24858233	chr3	**-**	-	OpenSea	1.272	1.84 × 10^−8^
cg11601932	chr3	**-**	-	OpenSea	0.645	3.52 × 10^−8^
cg27202913	chr16	CDH15	Body	Island	−1.055	5.45 × 10^−8^

**In bold:** genes enriched in normal breast tissue and breast cancer (CGAP); CGAP: Cancer Genome Anatomy Project; Chr—chromosome.

**Table 5:** CpG sites associated with breast cancer risk, passing the false discovery rate of 0.05 in the main analysis of normal breast tissue and replicated in the same direction in both secondary analyses (normal breast tissue and blood samples).

CpG	Chr	Gene	Gene Region	CpG Island Region	logFC	*p*-Value	*q*-Value
cg09434832	chr19	ZNF616	5′UTR	N_Shore	0.148	1.98 × 10^−5^	0.01
cg16927253	chr6	POM121L2	TSS1500	Island	−0.215	0.0001	0.02
cg13585675	chr7	**NPTX2**	Body	S_Shelf	0.289	0.0002	0.02
cg27639104	chr11	KCNQ1	1stExon;5′UTR;Body	OpenSea	0.181	0.0002	0.02
cg20754261	chr2	-	-	Island	−0.456	0.0006	0.04
cg03938369	chr12	CLEC4C *	Body	OpenSea	0.197	0.0006	0.04

**In bold:** genes enriched in breast cancer (CGAP); * genes enriched in the normal epithelium (CGAP); CGAP—Cancer Genome Anatomy Project; Chr—chromosome.

**Table 6:** Characteristics of the 20 most significantly differentially methylated regions (DMRs) in the main analysis of normal breast tissue, out of 96 significant DMRs.

Chr	Start Position	End Position	Width(bp)	N CpGs	Stouffer *p*-Value	Max Difference	Mean Difference	Overlapping Genes
chr19	58545001	58546307	1307	11	0.0000002	−0.056	−0.027	ZSCAN1
chr9	126775263	126778017	2755	9	0.0000025	−0.104	−0.041	**LHX2**
chr4	24796689	24797176	488	8	0.0000063	0.069	0.045	SOD3, SNORD74, SNORA3, snoR442, snoU2_19, SNORD65
chr8	145104971	145107199	2229	10	0.0000088	−0.099	−0.048	CTD-3065J16,6, OPLAH
chr5	115298079	115299828	1750	10	0.0000100	−0.057	−0.033	AQPEP, snoZ6, SNORA27, SNORA68, SNORA57, 7SK, SNORD45, SNORD95
chr17	42091713	42093050	1338	15	0.0000113	0.096	0.040	SNORA69, **TMEM101**
chr11	75139390	75139736	347	4	0.0000151	−0.096	−0.083	**KLHL35**
chr13	95357042	95359203	2162	14	0.0000217	0.091	0.051	SNORD36
chr19	58220080	58220837	758	11	0.0000255	−0.070	−0.044	ZNF551, AC003006,7, ZNF154
chr17	37365885	37366501	617	5	0.0000378	−0.071	−0.041	SNORA69
chr14	24422368	24423864	1497	10	0.0000452	0.081	0.026	**DHRS4-AS1**, **DHRS4**, SNORA79
chr1	203320190	203320541	352	6	0.0000549	−0.088	−0.060	snoU13, Y_RNA, SNORD112, U3, SNORA51, **SNORA25**, SCARNA20, SNORA70, SNORA77, SNORA26, SNORA72, U8, **FMOD**, SNORD60, SNORD116
chr15	34806349	34807143	795	5	0.0001300	−0.072	−0.030	**GOLGA8A**
chr19	18811575	18812017	443	4	0.0001515	0.062	0.046	**CRTC1**
chr16	50913892	50914024	133	3	0.0003178	−0.079	−0.068	SNORD111, SNORD33
chr6	50813341	50814305	965	6	0.0003740	−0.086	−0.046	SNORA38, SNORA8, SCARNA15, **TFAP2B**, SNORA20
chr5	102898223	102898729	507	5	0.0003923	−0.069	−0.038	snoZ6, SNORA27, SNORA68, SNORA57, 7SK, NUDT12, SNORD45, SNORD95
chr9	126784855	126785097	243	2	0.0005461	−0.063	−0.050	**LHX2**
chr4	6107021	6107791	771	8	0.0006567	−0.099	−0.043	SNORA3, **JAKMIP1**
chr8	144267518	144267725	208	2	0.0006981	−0.063	−0.045	-

**In bold**: genes also identified in the individual CpG analysis; DMR—differentially methylated regions; Chr—chromosome.

## Supplementary Materials

The following are available online at https://www.mdpi.com/2072-6694/12/11/3088/s1, Figure S1: Flow diagram representing data preprocessing methods and workflow for breast tissue samples, Figure S2: Flow diagram representing data preprocessing methods and workflow for blood samples, Table S1: Characteristics of patients with breast cancer (cases) and patients with a benign tumor (controls), in the secondary analysis of normal breast tissue, Table S2: Characteristics of patients who developed a primary breast cancer (cases) and patients who did not develop a primary breast cancer (controls), in the secondary analysis of blood samples, Table S3: Mean beta-values of patients with breast cancer (cases) and patients with a benign tumor (controls), in the secondary analysis of normal breast tissue, Table S4: Mean beta-values of patients who developed a primary breast cancer (cases) and patients who did not develop a primary breast cancer (controls), in the secondary analysis of blood samples, Table S5: Differentially methylated CpG sites in the main analysis of normal breast tissue, Table S6: Functional annotation analyses of the differentially methylated CpG sites in the main analysis of normal breast tissue, Table S7: Functional annotation analyses of the top 43 differentially methylated genes in the main analysis of normal breast tissue, Table S8: Differentially methylated CpG sites in the secondary analysis of normal breast tissue, Table S9: Differentially methylated CpG sites in the secondary analysis of blood tissue, Table S10: CpG sites associated with breast cancer risk, passing the false discovery rate of 0.05 in the main analysis of normal breast tissue and replicated in both secondary analyses (normal breast tissue and blood samples), Table S11: Differentially methylated regions associated with breast cancer risk in the main analysis of normal breast tissue, Table S12: Functional annotation analyses of differentially methylated regions associated with breast cancer risk in the main analysis of normal breast tissue.
