# Supplementary material for: DNA Methylation and Breast Cancer Risk: An Epigenome-Wide Study of Normal Breast Tissue and Blood
Source: Cancers (Basel). 2020 Oct 23;12(11):3088. doi: 10.3390/cancers12113088 (PMC7690691; doi:10.3390/cancers12113088)
Supplement: Supplementary file 1 [file cancers-12-03088-s001.zip › Supplementary files/cancers-933700-SI-Figure-Table1-4.docx]

**Supplementary Materials:**

DNA Methylation and Breast Cancer Risk: An Epigenome-Wide Study of Normal Breast Tissue and Blood

Kaoutar Ennour-Idrissi ^1,2,3^, Dzevka Dragic ^1,2^, Elissar Issa ^2,4^, Annick Michaud ^2^, Sue-Ling Chang ^2^, Louise Provencher ^5^, Francine Durocher ^2,4^ and Caroline Diorio ^1,2,5,^*


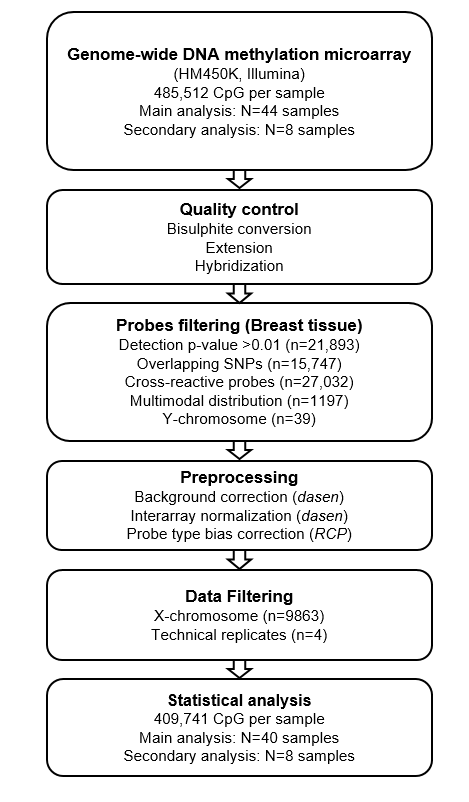


**Figure S1.**


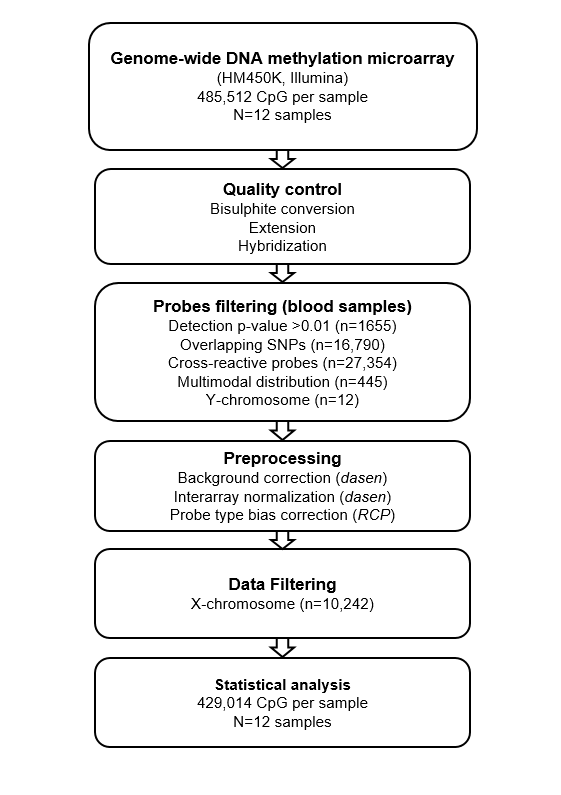


**Figure S2.**

**Table 1.** Characteristics of patients with breast cancer (cases) and patients with a benign tumor (controls), in the secondary analysis of normal breast tissue.

| **Characteristics** | **Cases (n=4)** | **Controls (n=4)** |
| --- | --- | --- |
| Age* (years) |  |  |
| Mean ± SD | 54.2 ± 7.3 | 56.7 ± 7.6 |
| Median [range] | 53.0 [47.0-64.0] | 56.5 [48.0-66.0] |
| Postmenopausal | 3 (75%) | 3 (75%) |
| Familial history of breast cancer (yes) | 1 (25%) | 4 (100%) |
| Parity (yes) | 3 (75%) | 4 (100%) |
| Age at first pregnancy (years) |  |  |
| Mean ± SD | 28.5 ± 7.8 | 24.5 ± 4.0 |
| Median [range] | 25.5 [23.0- 40.0] | 25.5 [19.0- 28.0] |
| Breastfeeding (yes) | 1 (25%) | 0 (0%) |
| Ever smokers | 1 (25%) | 1 (25%) |
| Alcohol consumption (yes) | 1 (25%) | 2 (50%) |
| Body mass index (kg/m^2^) |  |  |
| Mean ± SD | 23.3 ± 2.3 | 27.1 ± 5.2 |
| Median [range] | 24.2 [19.9-25.1] | 26.3 [22.1-33.6] |
| Histologic type |  |  |
| Ductal, invasive | 4 (100%) | - |
| Lobular, invasive | 0 | - |
| Grade |  |  |
| 1 | 0 | - |
| 2 | 3 (75%) | - |
| 3 | 1 (25%) | - |
| Stage |  |  |
| I | 3 (75%) | - |
| II | 1 (25%) | - |
| III | 0 | - |
| ER status |  |  |
| Negative | 0 | - |
| Positive | 4 (100%) | - |
| HER2 status |  |  |
| No evaluation | 0 | - |
| Negative | 4 (100%) | - |
| Positive | 0 | - |
| Chemotherapy | 2 (50%) | - |
| Radiotherapy | 3 (75%) | - |
| Hormone therapy | 4 (100%) | - |
| *at time of surgery; n= number; SD: standard deviation; -: Not applicable | | |

**Table 2.** Characteristics of patients who developed a primary breast cancer (cases) and patients who did not develop a primary breast cancer (controls), in the secondary analysis of blood samples.

| **Characteristics** | **Cases (n=6)** | **Controls (n=6)** |
| --- | --- | --- |
| Age^*^ (years) |  |  |
| Mean ± SD | 43.8 ± 5.5 | 43.6 ± 4.4 |
| Median [range] | 43.0 [38.0-53.0] | 43 [39.0-50.0] |
| Premenopausal^*^ | 6 (100%) | 6 (100%) |
| Familial history of breast cancer^*^ (yes) | 1 (16.7%) | 1 (16.7%) |
| Parity^*^ (yes) | 5 (83.3%) | 5 (83.3%) |
| Age at first pregnancy^*^ (years) |  |  |
| Mean ± SD | 28.2 ± 3.1 | 28.8 ± 2.6 |
| Median [range] | 27.0 [25.0-32.0] | 26.0 [22.0-29.0] |
| Breastfeeding^*^ (yes) | 5 (83.3%) | 2 (33.3%) |
| Ever smokers^*^ | 4 (66.7%) | 3 (50%) |
| Alcohol consumption^*^ (yes) | 6 (100%) | 6 (100%) |
| Body mass index^*^ (kg/m^2^) |  |  |
| Mean ± SD | 23.3 ± 2.5 | 25.3 ± 0.9 |
| Median [range] | 23.9 [18.8-25.6] | 24.9 [24.4-26.8] |
| Breast biopsy | 1 (16.7%) | 1 (16.7%) |
| Time from blood sample to cancer diagnosis (years) | | |
| Mean ± SD | 6.0 ± 1.6 | - |
| Median [range] | 6.0 [3.4-8.1] | - |
| Age at diagnosis (years) |  | - |
| Mean ± SD | 50.0 ± 6.1 |  |
| Median [range] | 48.2 [45.0-62.0] |  |
| Histologic type |  |  |
| Ductal, invasive | 6 (100%) | - |
| Lobular, invasive | 0 |  |
| Positive lymph nodes |  |  |
| 0 | 4 (66.6%) | - |
| 1-3 | 1 (16.7%) | - |
| ≥ 4 | 1 (16.7%) | - |
| Grade |  |  |
| 1 | 1 (16.7%) | - |
| 2 | 5 (83.3%) | - |
| 3 | 0 | - |
| ER status |  |  |
| Negative | 1 (16.7%) | - |
| Positive | 5 (83.3%) | - |
| HER2 status |  |  |
| No evaluation | 0 | - |
| Negative | 5 (83.3%) | - |
| Positive | 1 (16.7%) | - |
| Chemotherapy | 2 (33.3%) | - |
| Radiotherapy | 4 (66.7%) | - |
| Hormone therapy | 4 (66.7%) | - |
| *At time of blood sample; SD: standrad deviation; n= number; | | |

**Table 3.** Mean beta-values of patients with breast cancer (cases) and patients with a benign tumor (controls), in the secondary analysis of normal breast tissue.

|  | **Number of CpGs** | **Cases (n=4)** | **Controls (n=4)** | ***p*-value^*^** |
| --- | --- | --- | --- | --- |
| **All included CpGs** | 409,741 | 0.608 | 0.603 | 0.625 |
| **Distribution relative to island** | | | | |
| Island | 134,756 | 0.277 | 0.278 | 0.625 |
| N_Shelf | 19,957 | 0.735 | 0.733 | 0.625 |
| N_Shore | 53,628 | 0.511 | 0.509 | 0.625 |
| OpenSea | 141,767 | 0.699 | 0.696 | 0.625 |
| S_Shelf | 17,738 | 0.740 | 0.738 | 0.625 |
| S_Shore | 41,895 | 0.499 | 0.496 | 0.625 |
| **Distribution relative to gene** | | | | |
| TSS1500 | 72,345 | 0.385 | 0.384 | 0.875 |
| TSS200 | 55,782 | 0.244 | 0.245 | 0.625 |
| 5'UTR | 57,346 | 0.356 | 0.355 | 0.875 |
| 1stExon | 34,812 | 0.262 | 0.262 | 0.625 |
| Body | 150,226 | 0.630 | 0.628 | 0.375 |
| 3'UTR | 16,606 | 0.719 | 0.716 | 0.375 |
| Intergenic | 94,333 | 0.609 | 0.607 | 0.375 |
| Promoter | 83,922 | 0.178 | 0.178 | 0.875 |
| ^*^Wilcoxon signed-rank test; TSS : transcription start site | | | | |

**Table 4.** Mean beta-values of patients who developed a primary breast cancer (cases) and patients who did not develop a primary breast cancer (controls), in the secondary analysis of blood samples.

|  | **Number of CpGs** | **Cases (n = 6)** | **Controls (n = 6)** | ***p*-value^*^** |
| --- | --- | --- | --- | --- |
| **All included CpGs** | 429,014 | 0.519 | 0.520 | 0.313 |
| **Distribution relative to island** | | | | |
| Island | 136,512 | 0.245 | 0.245 | 0.563 |
| N_Shelf | 21,205 | 0.762 | 0.763 | 0.844 |
| N_Shore | 56,486 | 0.494 | 0.495 | 0.563 |
| OpenSea | 151,724 | 0.721 | 0.723 | 0.844 |
| S_Shelf | 18,938 | 0.767 | 0.769 | 0.844 |
| S_Shore | 44,149 | 0.486 | 0.487 | 1.000 |
| **Distribution relative to gene** | | | | |
| TSS1500 | 75,940 | 0.382 | 0.382 | 0.563 |
| TSS200 | 57,180 | 0.218 | 0.218 | 0.438 |
| 5'UTR | 59,555 | 0.348 | 0.349 | 0.563 |
| 1stExon | 35,360 | 0.240 | 0.240 | 0.438 |
| Body | 157,390 | 0.638 | 0.639 | 0.844 |
| 3'UTR | 17,573 | 0.739 | 0.741 | 0.844 |
| Intergenic | 99,869 | 0.612 | 0.614 | 0.844 |
| Promoter | 86,303 | 0.142 | 0.141 | 0.156 |
| ^*^Wilcoxon signed-rank test; TSS : transcription start site | | | | |
